# Underwater endoscopic papillectomy of a duodenal adenoma extending to the papilla using a forward-viewing endoscope

**DOI:** 10.1055/a-2616-8142

**Published:** 2025-06-26

**Authors:** Takashi Yamamoto, Yasushi Yamasaki, Yuki Fujii, Kazuyuki Matsumoto, Motoyuki Otsuka

**Affiliations:** 1 92057 Department of Gastroenterology, Okayama University Hospital, Okayama, Japan

Endoscopic resection of duodenal adenomas extending to the papilla is challenging 1 . Endoscopic papillectomy using an oblique-viewing endoscope is generally performed for ampullary adenomas; however, the vertical approach and snaring of the lesion carry a risk of muscle layer involvement, particularly in large lesions (>20 mm) or nonampullary adenomas extending to the papilla 2 . In contrast, a forward-viewing endoscope allows for a horizontal approach, enabling shallower resection and reducing the risk of perforation ( Fig. 1 ). We herein report a successful case of endoscopic papillectomy for a large duodenal adenoma extending to the papilla, performed using a forward-viewing endoscope in combination with the underwater technique ( Video 1 ).

**Fig. 1:**
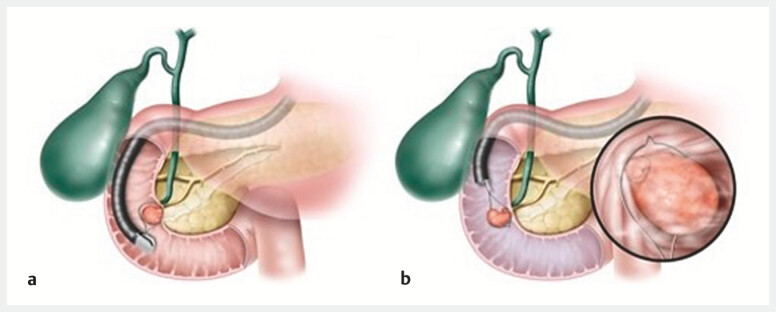
Schematic showing: **a** vertical snaring with an oblique-viewing endoscope; **b** horizontal snaring using a forward-viewing endoscope with the underwater technique.

Underwater endoscopic papillectomy is performed using a forward-viewing endoscope for a 25-mm duodenal adenoma extending to the papilla.Video 1

A woman in her 50s was referred to our hospital with a 25-mm duodenal adenoma extending to the papilla ( Fig. 2 **a** ). The lesion was primarily located on the distal side of the papilla, with extension to the papilla itself, posing a risk of intraoperative perforation if conventional endoscopic papillectomy with an oblique-viewing endoscope were performed. Therefore, we used a forward-viewing endoscope (PCF-290TI; Olympus, Tokyo, Japan). Underwater, the lesion floated owing to buoyancy and low intraduodenal pressure, facilitating easy snaring of the entire lesion with a horizontal approach. The lesion was resected en bloc using electrocautery, without any adverse events occurring ( Fig. 2 **b, c** ). After clip closure of the distal side of the mucosal defect had been completed, the scope was exchanged for an oblique-viewing endoscope (TJF-Q290V; Olympus), and a pancreatic stent was placed ( Fig. 2 **d** ). Histological examination confirmed a duodenal adenoma extending to the papilla, with negative horizontal and vertical margins; no tumor invasion was identified ( Fig. 3 ).

**Fig. 2:**
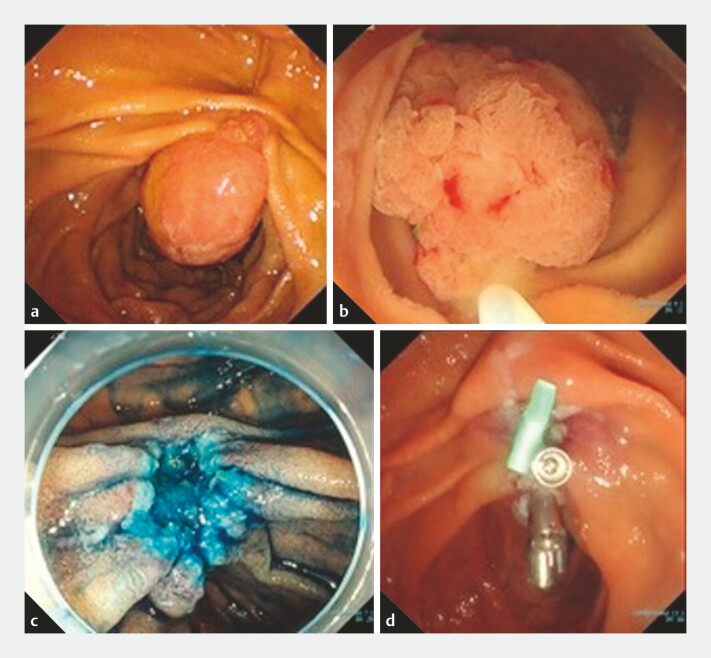
Endoscopic images during underwater endoscopic papillectomy using a forward-viewing endoscope showing: **a** a 25-mm duodenal adenoma involving the papilla of Vater (view with an oblique-viewing endoscope); **b** the lesion, including the papilla, snared during underwater endoscopic papillectomy (forward-viewing endoscope); **c** the mucosal defect after underwater endoscopic papillectomy stained with indigo carmine; **d** appearance after insertion of a pancreatic stent into the main pancreatic duct and closure of the mucosal defect with clips.

**Fig. 3:**
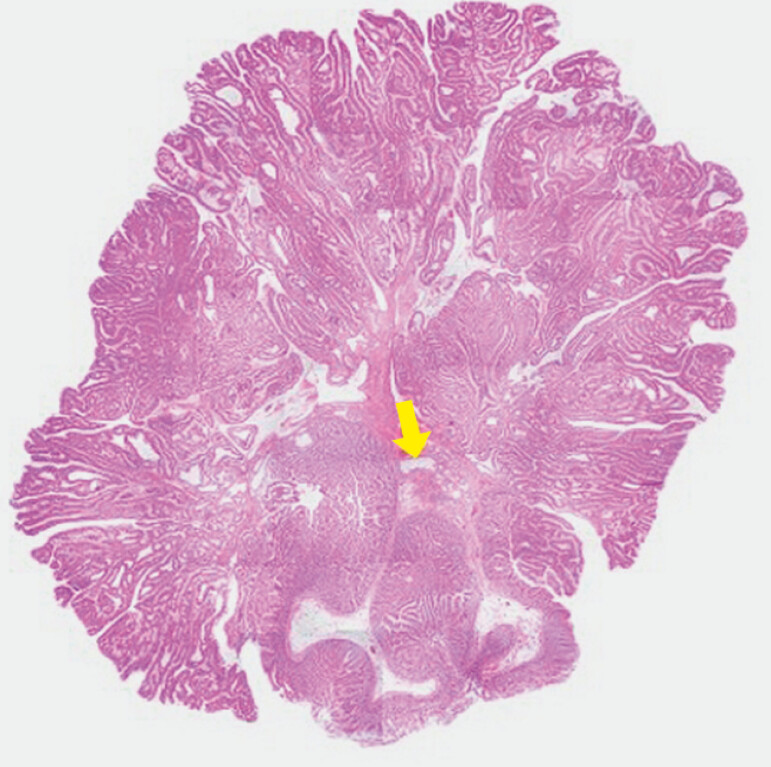
Histological examination of the resected lesion showing no tumor invasion into the main pancreatic duct (yellow arrow).

For ampullary adenomas, a vertical approach is crucial for deeper resection because of potential invasion into the bile and pancreatic ducts; however, a horizontal approach with a forward-viewing endoscope underwater may be more appropriate for duodenal adenomas extending to the papilla.

Endoscopy_UCTN_Code_TTT_1AR_2AF

## Notes

Endoscopy E-Videos https://eref.thieme.de/e-videos
*E-Videos* is an open access online section of the journal *Endoscopy* , reporting on interesting cases and new techniques in gastroenterological endoscopy. All papers include a high-quality video and are published with a Creative Commons CC-BY license. Endoscopy E-Videos qualify for HINARI discounts and waivers and eligibility is automatically checked during the submission process. We grant 100% waivers to articles whose corresponding authors are based in Group A countries and 50% waivers to those who are based in Group B countries as classified by Research4Life (see: https://www.research4life.org/access/eligibility/ ). This section has its own submission website at https://mc.manuscriptcentral.com/e-videos .
